# Cleaner outdoor air diminishes the overall risk of intracerebral hemorrhage but brings differential benefits to subpopulations: a time-stratified case-crossover study

**DOI:** 10.1186/s12889-023-16232-3

**Published:** 2023-07-07

**Authors:** Peng Wang, Wentao Feng, Shuang Luo, Shuwen Cheng, Min Gong, Yaxin Li, Yanhui Liu

**Affiliations:** 1 Department of Neurosurgery, West China Hospital, Sichuan University, Chengdu, China; 2 Department of Neurosurgery, Cancer Prevention and Treatment Institute of Chengdu, Chengdu Fifth People’s Hospital (The Second Clinical Medical College, Affiliated Fifth People’s Hospital of Chengdu University of Traditional Chinese Medicine), Chengdu, China; 3 West China Fourth Hospital/West China School of Public Health, Sichuan University, Chengdu, China

**Keywords:** Air pollution, Pollutant level, Short-term exposure, Intracerebral hemorrhage, Stroke, Disease risk

## Abstract

**Background:**

Short-term air pollution exposure and intracerebral hemorrhage (ICH) risk are related. However, the impact of the pollutant levels decline on this relationship, which attributes to clean air policy implementation and the COVID-19 pandemic lockdown, is unclear. In the present research, we explored the influence of different pollutant levels on ICH risk during eight years in a southwestern China megacity.

**Methods:**

Our research used a time-stratified case-crossover design. We retrospectively analyzed ICH patients in a teaching hospital from January 1, 2014, to December 31, 2021, and divided 1571 eligible cases into two groups (1st group: 2014–2017; 2nd group: 2018–2021). We observed the trend of every pollutant in the entire study period and compared the pollution levels in each group, using air pollutants data (PM_2.5_, PM_10_, SO_2_, NO_2_, CO, and O_3_) documented by the local government. We further established a single pollutant model via conditional logistic regression to analyze the association between short-term air pollutants exposure and ICH risk. We also discussed the association of pollution levels and ICH risk in subpopulations according to individual factors and monthly mean temperature.

**Results:**

We found that five air pollutants (PM_2.5_, PM_10_, SO_2_, NO_2_, CO) exhibited a continuous downward trend for the whole duration, and the daily concentration of all six pollutants decreased significantly in 2018–2021 compared with 2014–2017. Overall, the elevation of daily PM_2.5_, SO_2_, and CO was associated with increased ICH risk in the first group and was not positively associated with risk escalation in the second group. For patients in subgroups, the changes in the influence of lower pollutant levels on ICH risk were diverse. In the second group, for instance, PM_2.5_ and PM_10_ were associated with lower ICH risk in non-hypertension, smoking, and alcohol-drinking participants; however, SO_2_ had associations with increased ICH risk for smokers, and O_3_ had associations with raised risk in men, non-drinking, warm month population.

**Conclusions:**

Our study suggests that decreased pollution levels diminish the adverse effects of short-term air pollutants exposure and ICH risk in general. Nevertheless, the influence of lower air pollutants on ICH risk in subgroups is heterogeneous, indicating unequal benefits among subpopulations.

**Supplementary Information:**

The online version contains supplementary material available at 10.1186/s12889-023-16232-3.

## Background

Intracerebral hemorrhage (ICH), a dominant subtype of hemorrhagic stroke, is a leading cause of disability and death worldwide [1, 2]. According to The Global Burden of Diseases, Injuries, and Risk Factors Study, ICH constitutes 27.9% of all newly diagnosed strokes, and the proportion in World Bank low-income to upper-middle-income countries is nearly twice as high as that in high-income countries. Statistics on an international scale indicate that the absolute incidence of ICH is 34.1 per 100,000 annually, and the number of disability-adjusted life years is 685.7 per 100,000 [3]. In China, there were 0.85 million new ICH cases and 1.07 million related deaths in 2019, with a growth of 17.6% and 37.4% compared with 1990 [4]. The disease burden is severe nationally, [4] given that ICH patients always have poor prognoses and few proven treatments [5].

Increasing evidence demonstrates that air pollution is an influential environmental risk factor for stroke, and this risk factor is estimated to be responsible for 14% of all stroke-associated deaths [2]. Although not as numerous as studies for ischemic stroke, the association between short-term ambient air pollutants exposure and ICH risk is confirmed globally, including the countries in Asia, North America, and Europe [6–18]. In our previous report, we found that the daily concentration of particulate matter with an aerodynamic diameter ≤ 2.5 μm (PM_2.5_), particulate matter with an aerodynamic diameter ≤ 10 μm (PM_10_), sulfur dioxide (SO_2_), and carbon monoxide (CO) with a lag of 4 days had positive associations with the risk of ICH [15]. Therefore, it is logical to speculate that low pollutant level is conducive to reducing ICH risk and bringing benefits to this population.

Over the past decade, multinational governments have actively advocated clean air policies to control ambient air pollution. Particularly, since the ongoing COVID-19 global pandemic in 2019, the level of ambient pollutants has been an unprecedented drop due to the dramatic and sudden reduction in anthropogenic activity [19, 20]. There is a call for researchers to explore whether cleaner air can result in less air pollution-related disease, [2] although the executive air quality standards vary in different countries [21]. Studies to evaluate the impact of clean air on human diseases, such as cardiovascular disease and ischemic stroke, confirm significant reductions in air pollution-related total and cause-specific deaths and lower disease morbidity [22–24]. However, the investigation that analyzes the association between decreased short-term air pollutants exposure and ICH risk is scarce.

Chengdu is a megacity in southwestern China, having a subtropical monsoon climate and four distinct seasons, with low wind speed, high humidity, and less sunshine [25]. The outdoor pollutants detected by the local government since 2014 include PM_2.5_, PM_10_, SO_2_, nitrogen dioxide (NO_2_), CO, and ozone (O_3_). In the present research, we conducted a time-stratified case-crossover study to explore the relationship between ambient air pollutant levels and ICH risk over eight years in this region by dividing the participants into two groups. We also discussed the association of pollution levels and ICH risk in subpopulations according to individual factors and monthly mean temperature.

## Methods

### Study area and population

We retrospectively analyzed 1874 ICH patients in a teaching hospital (east longitude 103.8° and north latitude 30.7°) in the western region of Chengdu from January 1, 2014, to December 31, 2021 (2922 days). There were 1571 eligible cases with 303 exceptions. The hospital, the nearest medical center to the national benchmark climate station in Chengdu (straight-line distance = 6.4 km), covers an area of 277 square kilometers and provides medical services to a population of more than one million. We defined ICH cases as patients with I61 coding (ICD-10), which included cerebral hemisphere hemorrhage (lobar and deep), brainstem hemorrhage, cerebellar hemorrhage, ventricular hemorrhage, multi-site cerebral hemorrhage, and other cerebral hemorrhages. The diagnostic criteria for these participants refer to our previous report [15]. We excluded patients if they had macro-cerebrovascular diseases, attacks outside Chengdu, unclear onset time, or a lack of clinical data. All research procedures followed the ethical standards of the Chengdu Fifth People’s Hospital Ethical Committee (ref. no. 2019–085), and informed consent was waived since data were anonymized.

### Clinical, air pollution, and meteorological data

We collected clinical information of eligible cases using hospital electronic medical records. The patient's characteristics comprised age, sex, hypertension, diabetes, smoking, and alcohol drinking. Smoking history was defined as continuous or cumulative smoking for six months or more. Alcohol drinking history was defined as taking alcohol drink at least once a month or cumulative drinking for six months or more. We included six air pollutants (PM_2.5_, PM_10_, SO_2_, NO_2_, CO, and O_3_) and gathered their daily concentration from December 27, 2013, to December 31, 2021 (2927 days), utilizing the data from Chengdu Ecological and Environment Bureau (http://sthj.chengdu.gov.cn/). The daily values of PM_2.5_, PM_10_, SO_2_, and NO_2_ were the 24-h mean concentration, CO was the 95th percentile of the daily mean concentration, and O_3_ was the 90th percentile of the 8-h maximum concentration. In addition, we collected meteorological data of the same period, including daily temperature (TEM) and daily relative humidity (RHU), as covariables to control their influence on ICH risk. We also calculated the monthly mean TEM to determine the effect of the thermal background. The cold months were monthly mean TEM < 20 °C, and the warm months were ≥ 20 °C. The meteorological data were from the Chinese Meteorological Data Center (http://data.cma.cn/).

### Study design

**Fig. 1:**
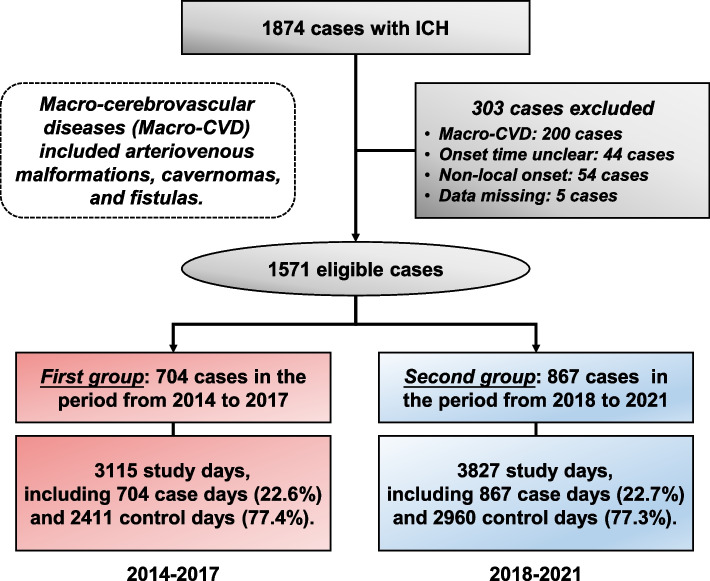
Flow chart of the present study. We retrospectively reviewed 1874 patients with intracerebral hemorrhage (ICH) and enrolled 1571 eligible cases with 303 exceptions. We divided the cases into two groups according to ICH onset time. Seven hundred and four patients belonged to the first group (red) from January 1, 2014, to December 31, 2017, and 867 belonged to the second (blue) from January 1, 2018, to December 31, 2021. Case days were the days when ICH symptoms occurred, and control days were corresponding days of the same month with seven days as the interval. Study days were the sum of case days and control days

Our study adopted a time-stratified case-crossover design. We divided eligible cases into two groups based on attack time. The first group included ICH patients from January 1, 2014, to December 31, 2017 (2014–2017), and the second from January 1, 2018, to December 31, 2021 (2018–2021) (Fig. 1). This time bisection can balance the number of cases in each study period, which helps to reduce selective bias. Case days (lag0) were the days when ICH symptoms occurred, and the control days were corresponding days of the same month with seven days as the interval. Study days were the sum of case days and control days. To analyze the lag effect, we included air pollution and meteorological data 1–5 days (lag1-5) before case days, and the selection of lag1-5 in the control days was accordant. The data we used were from the electronic medical record in the hospital, as the administration data can easily cause the dislocation classification of stroke time and shift the effect to zero [13].

### Statistical analysis

We plotted a scatter diagram with a locally weighted scatterplot smoothing curve for each air pollutant to assess the trend of the daily concentration level in the entire study period. We adopted the Chi-square test or Wilcoxon rank-sum test as appropriate for group comparison of the clinical, air pollution, and meteorological data and used Spearman correlation analysis for relationship exploration between the pollutants and meteorological data. We established a single pollutant model through conditional logistic regression to analyze the association between short-term air pollutants exposure and ICH risk in the two groups. The formula of the single-pollutant conditional logistic regression is as follows.

$$Logit\left({P}_{i}\right)=\mathrm{ln}\left(\frac{{P}_{i}}{1-{P}_{i}}\right)={\beta }_{0i}+{\beta }_{1}X+{\beta }_{2}TEM+{\beta }_{3}RHU$$

*P* was the probability of ICH occurrence, and *i* was the number of matched pairs. *X* represented a single air pollutant, for instance, PM_2.5_, PM_10_, SO_2_, NO_2_, CO, or O_3_*. β*_0i_ was the constant term of the matched pairs effect; *β*_1_, *β*_2_, and *β*_3_ were the model parameters.

We also investigated the influence of pollutants on ICH risk in different subpopulations by individual factors (age, sex, hypertension, diabetes, smoking, and alcohol drinking) and monthly mean TEM. Two variables did not enter regression analysis if their absolute value of correlation coefficient exceeded 0.9 [25]. We conducted sensitivity analyses to examine the robustness of our main results. (Supplementary materials) We calculated the odds ratios when the daily values of PM_2.5_, PM_10_, NO_2_, and O_3_ increased by 10 μg/m^3^, SO_2_ by 1 μg/m^3^, and CO by 0.1 mg/m^3^, respectively. Our research used R statistical software (4.1.2) and Graphpad (9.0) for analysis and plotting. The difference was statistically significant when *P* < 0.05.

## Results

### Statistical description

Our study enrolled 1571 ICH patients. The median age of the patients was 63 years (20 to 95), and nearly two-thirds were men (65.9%). 69.5% of the patients had hypertension, 11.7% had diabetes, and more than 30% had smoking (31.0%) or alcohol drinking (31.6%). According to the monthly mean TEM, 983 cases occurred in the cold month (< 20 °C), and the percentage was 62.6%. We divided the cases into two groups according to the onset time. There were 704 patients in the first group (2014–2017) and 867 in the second group (2018–2021). We did not observe a statistical difference in age, sex, diabetes, smoking, or alcohol drinking between these groups except for hypertension (Table 1).

**Table 1:** Baseline characteristics of patients with intracerebral hemorrhage enrolled in the present study

Variables	Overall (*n* = 1571)	Year: 2014–2017 (*n* = 704)	Year: 2018–2021 (*n* = 867)	*P *^***a***^
Age ^**b**^	63 (52, 73)	63 (51, 72)	64 (53, 74)	0.087
Sex				0.931
Male	1035 (65.9%)	463 (65.8%)	572 (66.0%)	
Female	536 (34.1%)	241 (34.2%)	295 (34.0%)	
Hypertension				0.019
Yes	1092 (69.5%)	468 (66.5%)	624 (72.0%)	
No	479 (30.5%)	236 (33.5%)	243 (28.0%)	
Diabetes				0.240
Yes	184 (11.7%)	75 (10.7%)	109 (12.6%)	
No	1387 (88.3%)	629 (89.3%)	758 (87.4%)	
Smoking				0.458
Yes	487 (31.0%)	225 (32.0%)	262 (30.2%)	
No	1084 (69.0%)	479 (68.0%)	605 (69.8%)	
Alcohol drinking				0.399
Yes	496 (31.6%)	230 (32.7%)	266 (30.7%)	
No	1075 (68.4%)	474 (67.3%)	601 (69.3%)	
Cold month				0.637
Yes	983 (62.6%)	436 (61.9%)	547 (63.1%)	
No	588 (37.4%)	268 (38.1%)	320 (36.9%)	

^***a***^Comparison was performed between patients in 2014–2017 and 2018–2021

^**b**^Data were expressed in median and upper/lower quartile

**Fig. 2:**
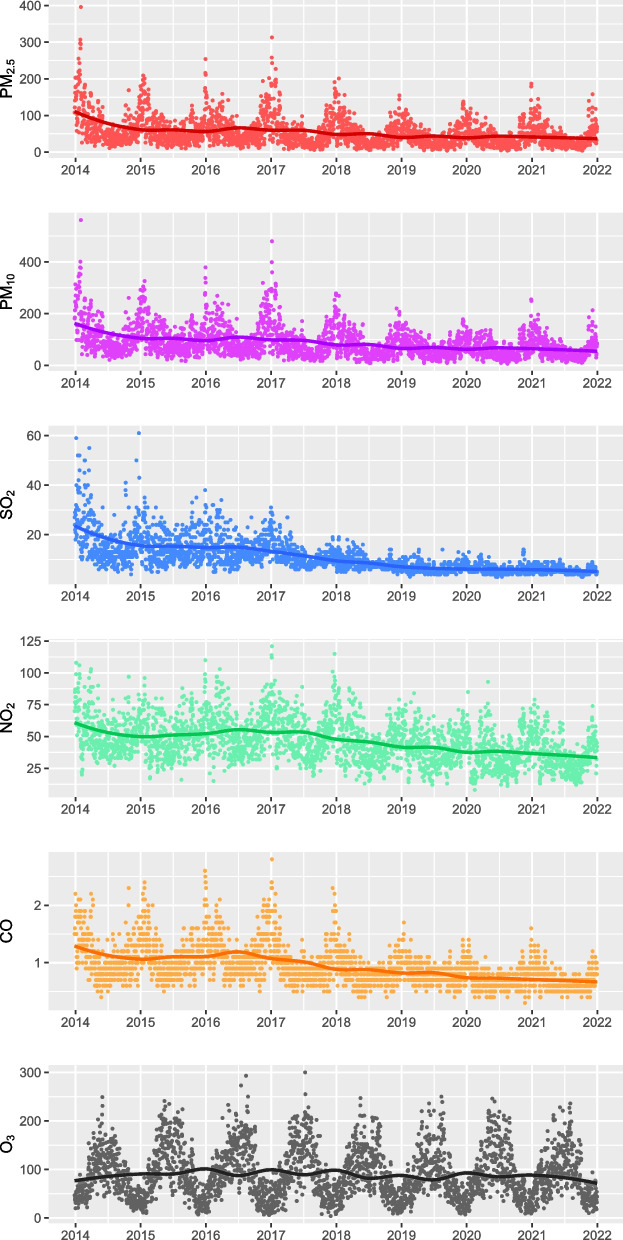
Scatter diagrams of daily air pollutants concentration with locally weighted scatterplot smoothing curves. The abscissa represents the date, and the ordinate represents the pollutant concentration. The unit of PM_2.5_, PM_10_, SO_2_, NO_2_, and, O_3_ is μg/m^3^, and the unit of CO is mg/m^3^

**Fig. 3:**
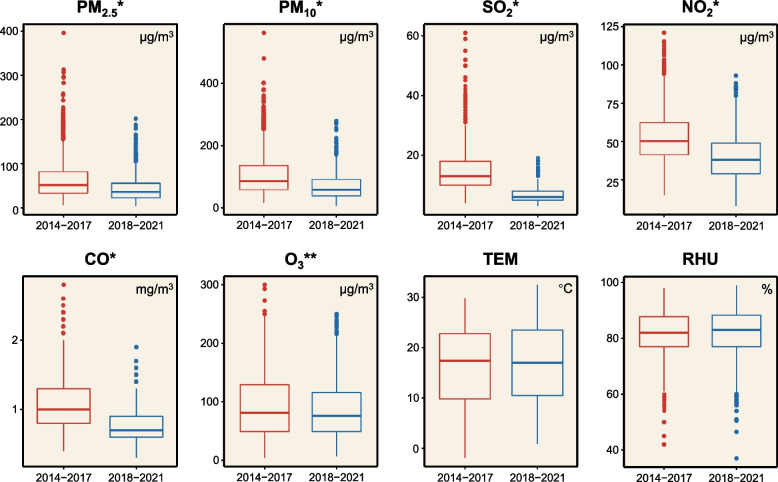
The box plot shows the comparison of air pollutants and meteorological parameters between 2014–2017 and 2018–2021. The thick line in the box represents the median (Q_2_), the upper bound of the box represents the upper quartile (Q_3_), and the lower bound of the box represents the lower quartile (Q_1_). The end of the line above the box represents the upper limit (Q3 + 1.5 × IQR), while the end of the line below the box represents the lower limit (Q1-1.5 × IQR). The points outside the box represent extreme values. * *P* < 0.01, **P < 0.05 Abbreviations: TEM, temperature; RHU, relative humidity; IQR, interquartile range

**Fig. 4:**
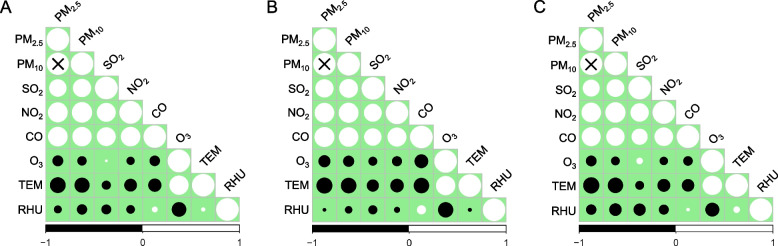
Spearman correlation analysis between air pollutants and meteorological parameters. The size of the circle indicates the absolute value of the correlation coefficient. The black circle represents a negative correlation coefficient, while the white circle represents a positive correlation coefficient. Symbol “ × ” represents the absolute value of the two variables’ correlation coefficient exceeding 0.9. **A**: Data from 2014 to 2021; **B**: Data from 2014 to 2017; **C**: Data from 2018 to 2021. Abbreviations: TEM, temperature; RHU, relative humidity

The scatter diagrams with locally weighted scatterplot smoothing curves demonstrate that five air pollutants (PM_2.5_, PM_10_, SO_2_, NO_2_, CO) exhibited a continuous downward trend, while O_3_ did not have this manifest tendency. (Fig. 2) Table 2 shows the statistical description of air pollutants and meteorological data in the two study groups (2014–2017 vs. 2018–2021). Compared with 2014–2017, the daily concentration of PM_2.5_, PM_10_, SO_2_, NO_2_, and CO in 2018–2021 decreased dramatically (*P* < 0.001). And the daily O_3_ level also declined significantly (*P* = 0.027), although not as encouraging as other pollutants. However, we did not observe a significant difference in daily TEM and RHU between the two periods (Fig. 3). The Spearman analysis showed that the absolute values of correlation coefficients of PM_2.5_ and PM_10_ were all greater than 0.9 in the entire (2014–2021), the first (2014–2017), and the second study period (2018–2021) (Fig. 4).

**Table 2:** Statistical descriptions of ambient air pollutants and meteorological parameters from December 27, 2013, to December 31, 2021, in Chengdu

Variables	2014–2017						2018–2021						*P *^***a***^
	Min	Q_1_	Q_2_	O_3_	Max	IQR	Min	Q_1_	Q_2_	O_3_	Max	IQR	
PM_2.5_ (μg/m^3^)	6.0	33.0	51.0	81.0	396.0	48.0	4.0	22.0	35.0	55.0	201.0	33.0	< 0.001
PM_10_ (μg/m^3^)	16.0	57.2	85.0	135.0	562.0	77.8	6.0	38.0	57.5	90.0	278.0	52.0	< 0.001
SO_2_ (μg/m^3^)	4.0	10.0	13.0	18.0	61.0	8.0	3.0	5.0	6.0	8.0	19.0	3.0	< 0.001
NO_2_ (μg/m^3^)	15.0	41.0	50.0	62.0	121.0	21.0	8.0	29.0	38.0	49.0	93.0	20.0	< 0.001
CO (mg/m^3^)	0.4	0.8	1.0	1.3	2.8	0.5	0.3	0.6	0.7	0.9	1.9	0.3	< 0.001
O_3_ (μg/m^3^)	4.0	49.0	81.0	129.0	300.0	80.0	7.0	49.0	76.0	116.0	250.0	67.0	0.027
TEM (◦C)	-1.9	9.8	17.4	22.8	29.8	13.0	0.9	10.5	17.0	23.5	32.5	13.0	0.257
RHU (%)	42.0	77.0	82.0	87.8	98.0	10.8	37.0	77.0	83.0	88.3	99.0	11.3	0.070

^**a**^Comparison was performed between 2014–2017 and 2018–2021 for air pollutants and meteorological parameters

Abbreviations: *TEM* Temperature, *RHU* Relative humidity, *Min* Minimum, *Q*_*1*_ Lower quartile, *Q2* Median, *Q*_*3*_ Upper quartile, *Max* Maximum, *IQR* Interquartile range

### Single pollutant model

**Fig. 5:**
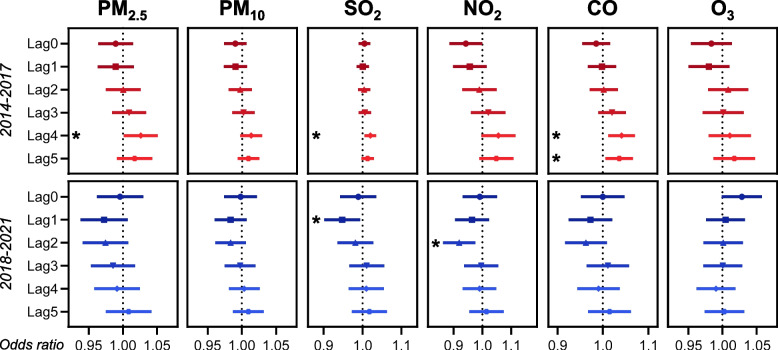
Forest plots of association between the increase of six air pollutants and intracerebral hemorrhage risk for cases in 2014–2017 and 2018–2021. Lag0 refers to the day of symptoms onset, lag1 refers to one day before lag0, lag2 refers to two days before lag0, so as lag3, lag4, and lag5. We calculated the odds ratios when the daily values of PM_2.5_, PM_10_, NO_2_, and O_3_ increased by 10 μg/m^3^, SO_2_ by 1 μg/m^3^, and CO by 0.1 mg/m.^3^, respectively. * *P* < 0.05

We constructed a single pollutant model to study the association between ambient air pollutants and the ICH risk in each group. The results revealed that the elevation of daily concentration of PM_2.5_ (lag4), SO_2_ (lag4), and CO (lag4&5) was associated with increased ICH risk in the first group. Nevertheless, for the cases in the second group, the increment of pollutants was not positively associated with risk escalation. Unexpectedly, the increase of SO_2_ (lag1) and NO_2_ (lag2) in the second group was even associated with decreased ICH risk (Fig. 5).

### Stratified analysis

**Fig. 6:**
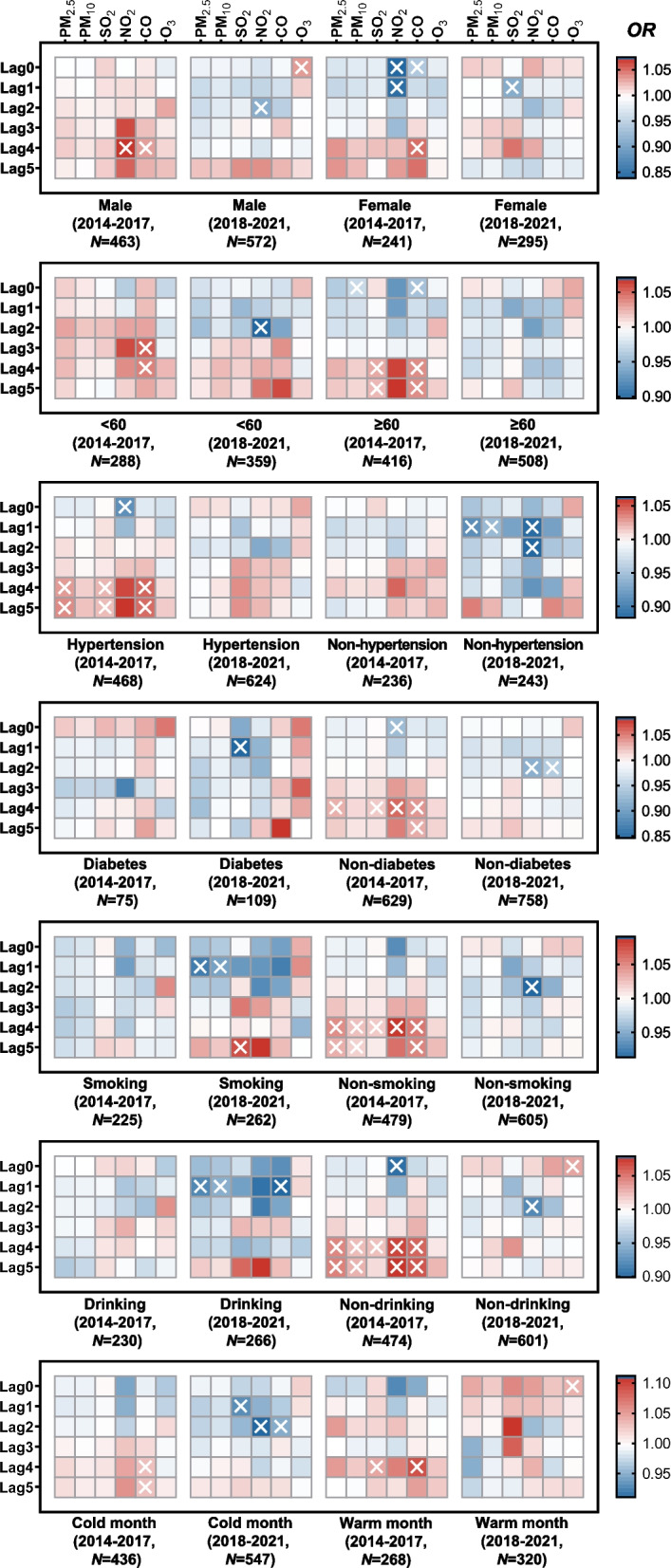
Odds ratio (OR) of intracerebral hemorrhage risk associated with the increase of six air pollutants in subgroups stratified by individual factors (age, sex, hypertension, diabetes, smoking, and alcohol drinking) and monthly mean temperature in 2014–2017 and 2018–2021. Lag0 refers to the day of symptoms onset, lag1 refers to one day before lag0, lag2 refers to two days before lag0, so as lag3, lag4, and lag5. We calculated the odds ratios when the daily values of PM_2.5_, PM_10_, NO_2_, and O_3_ increased by 10 μg/m^3^, SO_2_ by 1 μg/m^3^, and CO by 0.1 mg/m.^3^, respectively. Symbol “ × ” represents *P* < 0.05

We further conducted a stratified analysis, and the results showed various associations between air pollutants and ICH risk in subgroup patients (Fig. 6). The detailed results are as follows.

1. In the first group, PM_2.5_ and PM_10_ were associated with increased ICH risk for non-smoking or non-drinking patients, and PM_2.5_ was associated with an increased risk of cases with hypertension or non-diabetes. However, PM_10_ had associations with decreased risk for elderly patients (≥ 60). In the second group, PM_2.5_ and PM_10_ had associations with lowered risk in non-hypertension, smoking, or alcohol-drinking cases.
2. SO_2_ in the first group was associated with increased ICH risk for the old (≥ 60), hypertension, non-diabetes, non-smoking, non-drinking, or warm month population. And SO_2_ in the second group had associations with a decreased risk for females, diabetes, or cold month subgroups. But, SO_2_ was associated with increased risk for smokers in the second group.
3. NO_2_ in the first group was associated with increased ICH risk in men or non-smokers. While NO_2_ had associations with decreased risk for women or hypertension patients in the first group, and this pollutant was also associated with lowered risk for males, youth (< 60), non-hypertension, non-diabetes, non-smoking, non-drinking, or cold month population in the second group. Surprisingly, for non-diabetes or non-drinking cases in the first group, NO_2_ had different relationships with ICH risk in diverse lag days.
4. CO was associated with increased ICH risk for men, youth (< 60), hypertension, non-diabetes, non-smoking, non-drinking, cold, or warm month population in the first group, and CO had associations with reduced risk for non-diabetes, alcohol drinking, or cold month patients in the second group. CO had different effects on ICH risk in female or elderly patients (≥ 60) in the first group at different lag stages.
5. O_3_ was only associated with increased ICH risk in men, non-drinking, or warm month population of the second group.

## Discussion

In this study, we explored the impact of different pollutant levels on the ICH risk in totality and subpopulation from 2014 to 2021, considering the decline in pollutant levels caused probably by the local government’s clean air policy and the lockout since the covid-19 global pandemic in 2019. We only focused on ICH patients as previous studies concerning the association between air pollution and stroke risk usually did not distinguish ischemic stroke from hemorrhagic stroke, which led to challenges in interpreting the result [2]. Our main findings were: 1) the ambient air pollutants levels decreased during 2018–2021, 2) the reduction in air pollutants, including PM_2.5_, SO_2_, and CO, weakened their adverse association with total ICH risk, 3) and the effect of lower pollutants levels on ICH risk in subgroups was heterogeneous.

The daily concentrations of six dominant air pollutants significantly decreased in 2018–2021 compared with 2014–2017 in Chengdu. According to WHO Global Air Quality Guidelines, [26] the medians of PM_2.5_, PM_10_, and NO_2_ reached higher interim targets in 2018–2021 than in 2014–2017, and the medians of SO_2_, CO, and O_3_ matched air quality guideline levels in both study periods. These results indicate that the air quality level in the city has remarkably improved. One possible reason may be related to the local government's successive measures to improve the ecological environment, including continuous strengthening of comprehensive management of air, water, and soil pollution (http://www.scaepp.cn/sthjt/index.shtml). Another possible reason may be the lockdown since the COVID-19 global pandemic, accompanied by notable reductions in industrial and vehicle emissions of air pollutants [19, 20]. In addition, we found that five air pollutants (PM_2.5_, PM_10_, SO_2_, NO_2_, CO) exhibited a continuous downward trend in the entire period. This result suggests a persistent improvement in air quality in the region we are studying, although the COVID-19 pandemic lockdown may accelerate this process.

Particulate matter (PM) is the most commonly studied pollutant in exploring the biological effect of air pollution on health. Compared with gaseous pollutants, PM has more toxic effects on humans [27]. Previous research shows that the relationship between PM and ICH risk is inconsistent. Studies in heavily polluted areas reveal that PM significantly increases ICH risk, [11, 15] but others in some areas with low-level air pollution indicate that PM is irrelevant to ICH risk or even has a negative association [12, 13, 17]. We found that PM_2.5_ was associated with increased ICH risk of entire participants in the first group when the daily concentration of pollutants was at a high level. However, it was not related to the ICH risk in the second group when the pollutant level decreased. These outcomes suggest that decreased pollution levels lessen the adverse biological association between short-term PM_2.5_ exposure and ICH risk. We did not observe a similar phenomenon for PM_10_ in the present study, which may be because this pollutant is less toxic than PM_2.5_ [6].

Our study also demonstrated that PM, including PM_2.5_ and PM_10_, was associated with decreased ICH risk in some subpopulations, and this mainly occurred within the second group with lower pollutant levels. We speculate that PM may play a "double role" on cerebral vessels in some ICH subpopulations, as PM in previous research can increase the risk of ischemic and hemorrhagic stroke, respectively [11, 15, 28]. A potential explanation for detail is that the effect of PM causing a cerebral vascular rupture and bleeding is dominant when air pollution is heavy. However, the phenomenon that PM compels cerebrovascular thrombosis becomes a leading factor when the level of pollutants decreases, which lowers the bleeding risk. Nonetheless, it is critical to note that our hypothesis of the dual influence of PM on cerebral vessels needs confirmation by further studies. In addition, we discovered that PM_10_ was associated with decreased risk for elderly patients (≥ 60) in the first group. A possible reason is that the elderly patients in the present study may have less time to be exposed to the outdoor environment, thus showing a low biological sensitivity.

SO_2_, a colorless acid gas, is a common air pollutant. Our study found that SO_2_ had associations with increased ICH risk when the daily average concentration level was remarkable, and this positive effect changed in the opposite direction with a descending ICH risk when the SO_2_ level dropped. Previous studies also suggest that SO_2_ can boost ICH risk in areas with significant air pollution, [9] and this pollutant is related to ICH risk reduction within an environment with relatively low SO_2_ concentration [17]. These results display that SO_2_ exposure at different levels causes distinct ICH risk changes. We think the possible reason may be as SO_2_, similar to PM, may also have a two-way effect on causing an ischemic and hemorrhagic stroke. Interestingly, in stratified analysis, low concentrations of SO_2_ had associations with increased ICH risk in smoking patients, which may be due to some synergy mechanism.

NO_2_ is another prevailing acid gas pollutant. We discovered that this pollutant was associated with a reduced ICH risk in total participants when the daily concentration decreased. A nationwide study from Singapore found a similar result, which suggests that NO_2_ with a low level is negatively associated with ICH risk in the Asian population [17]. Nevertheless, we observed that the relatively high daily concentration of NO_2_ was associated with decreased ICH risk for women and patients with hypertension, and NO_2_ showed opposite effects in different lag periods for patients without diabetes and alcohol consumption. We speculated that this phenomenon may be due to the distinctions in lag effects between air pollutants. Further study is necessary to explore this diverse influence of NO_2_ on ICH risk in distinct subpopulations.

CO is a neutral gas, which is less studied when discussing the impact of air pollution on ICH risk. The findings of two studies from Asia indicate that the increase in daily CO concentration aggravates ICH risk in the condition of short-term exposure [15, 17]. In the present research, CO had associations with increased ICH risk when the concentration level was high. Nonetheless, the effect disappeared when the pollution level dropped. This result suggests that a decrease in CO level helps reduce its positive association with ICH risk. In stratified analysis, CO at a high level showed opposite effects in different lag periods for females and ≥ 60 patients, which may be caused by the differences in pollutant exposure between participants and lag effects between air pollutants.

We did not observe that O_3_ was associated with increased ICH risk in the overall case, regardless of the pollutant level. However, in subgroups (men, non-drinkers) and warm months, low concentration of O_3_ has associations with increased ICH risk. Nhung and their colleagues also discovered that the effect of O_3_ is weaker in cold seasons compared with other pollutants [29]. This outcome may be interpreted by the diversity in exposure between populations and the unbalanced seasonal distribution of O_3_.

Our research highlights the influence of ambient air pollution on ICH risk by comparatively studying two patient groups with different air pollution exposure levels. There are several limitations. 1) The data in this study came from a single city. And the results may not be generalized to ICH patients in other areas, given that the climatic characteristics and specific components of pollutants, such as PM_2.5_, may vary from region to region. A multicenter large-sample study on the impact of declining pollutant levels on ICH risk is essential. 2) Our study did not include indoor air pollutants, which also can increase the risk of ICH. Especially during the COVID-19 epidemic, indoor pollutants exposure may increase because people spend more time indoors due to the lockdown. Further studies are needed to clarify the association between low indoor pollutant levels and ICH risk. 3) We did not explore the influence of long-term air pollution exposure on ICH risk. Previous studies have shown that long-term exposure to pollutants significantly promotes the risk of stroke [30, 31]. Considering the cumulative effect, the assumption that long-term exposure still has an adverse association with health may be reasonable, even if the level of pollutants decreases significantly.

## Conclusions

Our study suggests that decreased pollution levels diminish the adverse effects of short-term ambient air pollutants exposure on ICH risk in general, which demonstrates that clean air policies with the effort to lower pollution levels have positive preventive significance. Nevertheless, the influence of lower air pollutants on ICH risk in subgroups with different individual risks or monthly mean temperature is heterogeneous, indicating differential benefits among subpopulations.

## Supplementary Information

**Additional file 1.**

## Data Availability

The datasets generated and/or analyzed during the current study are available in the Chengdu Ecology and Environment Bureau and the Chinese Meteorological Data Center. Chengdu Ecology and Environment Bureau:http://sthj.chengdu.gov.cn/ Chinese Meteorological Data Center:http://data.cma.cn/

## Abbreviations

ICH: Intracerebral hemorrhage
PM_2.5_: Particulate matter with an aerodynamic diameter ≤ 2.5 μm
PM_10_: Particulate matter with an aerodynamic diameter ≤ 10 μm
SO_2_: Sulfur dioxide
NO_2_: Nitrogen dioxide
CO: Carbon monoxide
O_3_: Ozone
TEM: Temperature
RHU: Relative humidity
PM: Particulate matter
OR: Odds ratio
Min: Minimum
Max: Maximum
IQR: Interquartile range
