# Supplementary material for: Cleaner outdoor air diminishes the overall risk of intracerebral hemorrhage but brings differential benefits to subpopulations: a time-stratified case-crossover study
Source: BMC Public Health. 2023 Jul 7;23:1303. doi: 10.1186/s12889-023-16232-3 (PMC10327021; doi:10.1186/s12889-023-16232-3)
Supplement: Supplementary file 1 — Additional file 1. [file 12889_2023_16232_MOESM1_ESM.docx]

**SUPPLEMENTARY MATERIALS**

| ***Contents***  [**Sensitivity Analyses** 1](#_Toc135143553)  [**Supplementary Figure 1** *The referent schemes of time-stratified case-crossover and symmetric bidirectional case-crossover design* 2](#_Toc135143554)  [**Supplementary Table 1** *The association of ambient air pollutants and intracerebral hemorrhage risk in a single pollutant model adjusted for TEM and/or RHU (OR, 95% CI)* 3](#_Toc135143555)  [**Supplementary Table 2** *The association of ambient air pollutants and intracerebral hemorrhage risk in time-stratified case-crossover design and symmetric bidirectional case-crossover design (OR, 95% CI)* 6](#_Toc135143556) |
| --- |

**Sensitivity Analyses**

We conducted sensitivity analyses to examine the robustness of our main results. Firstly, considering the possible covariate effect, we only included a single air pollutant and a single covariate (temperature or humidity) for conditional logistic regression analysis. The result indicated that the elevation of daily PM_2.5_, SO_2_, and CO was still associated with increased ICH risk in the first group and was not positively associated with risk escalation in the second group when separately adjusted for temperature or humidity. (**Supplementary Table 1**) Secondly, we compared the difference between the two ways to identify controls in the case-crossover design (time-stratified case-crossover vs. symmetric bidirectional case-crossover, **Supplementary Figure 1**). The outcome revealed that SO_2_ and CO had associations with increased ICH risk in the first group when using the symmetric bidirectional case-crossover design. PM_2.5_ did not have associations with increased ICH risk, which is probably due to the differences between these two methods. (**Supplementary Table 2**)





**Supplementary Figure 1** The referent schemes of time-stratified case-crossover and symmetric bidirectional case-crossover design. The red circle represents the case days (the day of intracerebral hemorrhage onset), while the black circle represents the control days.

**Supplementary Table 1** The association of ambient air pollutants and intracerebral hemorrhage risk in a single pollutant model adjusted for TEM and/or RHU (OR, 95% CI)

|  | Adjusted for TEM  and RHU | Adjusted for TEM | Adjusted for RHU |
| --- | --- | --- | --- |
| PM_2.5_ | | | |
| Year: 2014-2017 | | | |
| Lag 0 | 0.989 (0.964, 1.015) | 0.991 (0.966, 1.016) | 0.979 (0.955, 1.004) |
| Lag 1 | 0.989 (0.963, 1.016) | 0.992 (0.967, 1.017) | 0.982 (0.958, 1.008) |
| Lag 2 | 1.000 (0.975, 1.026) | 1.000 (0.975, 1.025) | 0.998 (0.973, 1.023) |
| Lag 3 | 1.009 (0.984, 1.034) | 1.008 (0.984, 1.032) | 1.010 (0.986, 1.034) |
| Lag 4 | **1.026 (1.001, 1.051) *** | **1.027 (1.003, 1.052) *** | **1.026 (1.002, 1.051) *** |
| Lag 5 | 1.017 (0.991, 1.043) | 1.018 (0.993, 1.043) | 1.017 (0.992, 1.042) |
| Year: 2018-2021 | | | |
| Lag 0 | 0.995 (0.962, 1.030) | 0.993 (0.960, 1.027) | 0.983 (0.951, 1.017) |
| Lag 1 | 0.972 (0.938, 1.007) | 0.970 (0.936, 1.004) | **0.962 (0.930, 0.996) *** |
| Lag 2 | 0.974 (0.941, 1.008) | 0.975 (0.942, 1.008) | 0.972 (0.940, 1.004) |
| Lag 3 | 0.985 (0.953, 1.018) | 0.985 (0.953, 1.018) | 0.983 (0.952, 1.015) |
| Lag 4 | 0.991 (0.958, 1.025) | 0.990 (0.958, 1.024) | 0.989 (0.958, 1.022) |
| Lag 5 | 1.008 (0.975, 1.042) | 1.007 (0.975, 1.041) | 1.007 (0.976, 1.040) |
| PM_10_ |  |  |  |
| Year: 2014-2017 | | | |
| Lag 0 | 0.990 (0.973, 1.006) | 0.990 (0.974, 1.007) | 0.984 (0.968, 1.000) |
| Lag 1 | 0.990 (0.973, 1.007) | 0.991 (0.974, 1.008) | 0.986 (0.970, 1.003) |
| Lag 2 | 0.997 (0.980, 1.014) | 0.997 (0.980, 1.013) | 0.996 (0.979, 1.012) |
| Lag 3 | 1.002 (0.985, 1.018) | 1.001 (0.985, 1.018) | 1.002 (0.987, 1.018) |
| Lag 4 | 1.013 (0.997, 1.029) | 1.014 (0.998, 1.030) | 1.013 (0.998, 1.030) |
| Lag 5 | 1.009 (0.993, 1.025) | 1.009 (0.993, 1.026) | 1.009 (0.993, 1.025) |
| Year: 2018-2021 | | | |
| Lag 0 | 0.998 (0.974, 1.022) | 0.998 (0.974, 1.022) | 0.990 (0.967, 1.013) |
| Lag 1 | 0.983 (0.960, 1.007) | 0.984 (0.961, 1.008) | 0.977 (0.955, 1.000) |
| Lag 2 | 0.983 (0.961, 1.006) | 0.983 (0.961, 1.006) | 0.981 (0.959, 1.004) |
| Lag 3 | 0.997 (0.975, 1.020) | 0.997 (0.975, 1.020) | 0.995 (0.974, 1.017) |
| Lag 4 | 1.003 (0.981, 1.026) | 1.003 (0.981, 1.026) | 1.002 (0.980, 1.024) |
| Lag 5 | 1.009 (0.987, 1.032) | 1.009 (0.987, 1.032) | 1.009 (0.987, 1.031) |

***** *P*<0.05

**Abbreviations:** OR, odds ratio; CI, confidence interval; TEM, temperature; RHU, relative humidity

***Continued***

|  | Adjusted for TEM  and RHU | Adjusted for TEM | Adjusted for RHU |
| --- | --- | --- | --- |
| SO_2_ | | | |
| Year: 2014-2017 | | | |
| Lag 0 | 1.005 (0.990, 1.020) | 1.004 (0.989, 1.019) | 1.000 (0.985, 1.015) |
| Lag 1 | 1.001 (0.985, 1.016) | 1.000 (0.985, 1.016) | 0.997 (0.982, 1.013) |
| Lag 2 | 1.004 (0.989, 1.020) | 1.004 (0.989, 1.020) | 1.003 (0.988, 1.019) |
| Lag 3 | 1.006 (0.990, 1.022) | 1.006 (0.990, 1.022) | 1.006 (0.991, 1.022) |
| Lag 4 | **1.020 (1.005, 1.035) *** | **1.019 (1.004, 1.034) *** | **1.020 (1.005, 1.035) *** |
| Lag 5 | 1.013 (0.997, 1.029) | 1.012 (0.996, 1.028) | 1.013 (0.997, 1.028) |
| Year: 2018-2021 | | | |
| Lag 0 | 0.988 (0.943, 1.036) | 0.995 (0.950, 1.041) | 0.968 (0.925, 1.012) |
| Lag 1 | **0.947 (0.902, 0.994) *** | 0.954 (0.910, 1.001) | **0.934 (0.892, 0.979) *** |
| Lag 2 | 0.981 (0.936, 1.028) | 0.982 (0.938, 1.028) | 0.976 (0.934, 1.021) |
| Lag 3 | 1.010 (0.966, 1.056) | 1.011 (0.967, 1.056) | 1.005 (0.963, 1.050) |
| Lag 4 | 1.009 (0.965, 1.055) | 1.010 (0.967, 1.055) | 1.005 (0.963, 1.050) |
| Lag 5 | 1.017 (0.973, 1.063) | 1.017 (0.973, 1.062) | 1.016 (0.974, 1.060) |
| NO_2_ |  |  |  |
| Year: 2014-2017 | | | |
| Lag 0 | 0.942 (0.886, 1.001) | 0.943 (0.887, 1.002) | **0.926 (0.873, 0.983) *** |
| Lag 1 | 0.956 (0.899, 1.016) | 0.957 (0.900, 1.017) | 0.946 (0.891, 1.005) |
| Lag 2 | 0.989 (0.931, 1.051) | 0.988 (0.930, 1.050) | 0.985 (0.928, 1.047) |
| Lag 3 | 1.020 (0.961, 1.082) | 1.020 (0.961, 1.082) | 1.021 (0.963, 1.083) |
| Lag 4 | 1.055 (0.997, 1.117) | 1.056 (0.998, 1.117) | 1.057 (0.999, 1.118) |
| Lag 5 | 1.048 (0.990, 1.110) | 1.049 (0.990, 1.111) | 1.048 (0.990, 1.110) |
| Year: 2018-2021 | | | |
| Lag 0 | 0.990 (0.932, 1.052) | 0.994 (0.936, 1.056) | 0.973 (0.917, 1.033) |
| Lag 1 | 0.963 (0.905, 1.024) | 0.967 (0.909, 1.028) | 0.948 (0.893, 1.007) |
| Lag 2 | **0.918 (0.864, 0.976) *** | **0.919 (0.864, 0.977) *** | **0.916 (0.862, 0.973) *** |
| Lag 3 | 0.995 (0.937, 1.056) | 0.996 (0.938, 1.057) | 0.991 (0.935, 1.051) |
| Lag 4 | 0.989 (0.932, 1.049) | 0.990 (0.933, 1.050) | 0.987 (0.931, 1.046) |
| Lag 5 | 1.013 (0.955, 1.075) | 1.013 (0.955, 1.075) | 1.013 (0.955, 1.074) |

***** *P*<0.05

**Abbreviations:** OR, odds ratio; CI, confidence interval; TEM, temperature; RHU, relative humidity

***Continued***

|  | Adjusted for TEM  and RHU | Adjusted for TEM | Adjusted for RHU |
| --- | --- | --- | --- |
| CO | | | |
| Year: 2014-2017 | | | |
| Lag 0 | 0.985 (0.955, 1.016) | 0.988 (0.960, 1.018) | 0.973 (0.945, 1.003) |
| Lag 1 | 0.998 (0.967, 1.029) | 1.001 (0.972, 1.030) | 0.989 (0.960, 1.020) |
| Lag 2 | 1.002 (0.971, 1.033) | 1.001 (0.972, 1.030) | 0.999 (0.969, 1.029) |
| Lag 3 | 1.020 (0.990, 1.051) | 1.018 (0.989, 1.048) | 1.021 (0.991, 1.051) |
| Lag 4 | **1.041 (1.012, 1.071) *** | **1.042 (1.014, 1.070) *** | **1.042 (1.013, 1.071) *** |
| Lag 5 | **1.036 (1.006, 1.066) *** | **1.035 (1.007, 1.064) *** | **1.035 (1.006, 1.065) *** |
| Year: 2018-2021 | | | |
| Lag 0 | 0.999 (0.952, 1.048) | 0.994 (0.948, 1.042) | 0.980 (0.935, 1.026) |
| Lag 1 | 0.972 (0.925, 1.021) | 0.966 (0.920, 1.014) | 0.956 (0.912, 1.002) |
| Lag 2 | 0.962 (0.917, 1.009) | 0.955 (0.911, 1.000) | **0.950 (0.907, 0.994) *** |
| Lag 3 | 1.010 (0.965, 1.058) | 1.009 (0.964, 1.055) | 1.005 (0.962, 1.050) |
| Lag 4 | 0.990 (0.944, 1.037) | 0.988 (0.944, 1.034) | 0.987 (0.944, 1.032) |
| Lag 5 | 1.014 (0.968, 1.062) | 1.013 (0.969, 1.059) | 1.013 (0.969, 1.058) |
| O_3_ |  |  |  |
| Year: 2014-2017 | | | |
| Lag 0 | 0.983 (0.954, 1.014) | 0.985 (0.960, 1.011) | **0.967 (0.941, 0.995) *** |
| Lag 1 | 0.980 (0.950, 1.010) | 0.982 (0.956, 1.008) | **0.970 (0.944, 0.998) *** |
| Lag 2 | 1.008 (0.979, 1.038) | 1.007 (0.981, 1.033) | 1.002 (0.975, 1.030) |
| Lag 3 | 1.001 (0.971, 1.031) | 1.001 (0.976, 1.027) | 1.003 (0.976, 1.032) |
| Lag 4 | 1.010 (0.980, 1.042) | 1.002 (0.976, 1.028) | 1.012 (0.983, 1.042) |
| Lag 5 | 1.017 (0.987, 1.048) | 1.009 (0.983, 1.036) | 1.016 (0.988, 1.046) |
| Year: 2018-2021 | | | |
| Lag 0 | 1.028 (0.999, 1.058) | **1.030 (1.003, 1.057) *** | 0.999 (0.975, 1.024) |
| Lag 1 | 1.004 (0.976, 1.033) | 1.012 (0.986, 1.038) | 0.987 (0.963, 1.011) |
| Lag 2 | 1.001 (0.972, 1.030) | 1.001 (0.975, 1.027) | 0.994 (0.970, 1.019) |
| Lag 3 | 1.000 (0.972, 1.029) | 1.002 (0.976, 1.028) | 0.996 (0.972, 1.020) |
| Lag 4 | 0.990 (0.962, 1.019) | 0.994 (0.968, 1.020) | 0.989 (0.966, 1.014) |
| Lag 5 | 1.002 (0.974, 1.032) | 1.002 (0.976, 1.028) | 1.002 (0.978, 1.027) |

***** *P*<0.05

**Abbreviations:** OR, odds ratio; CI, confidence interval; TEM, temperature; RHU, relative humidity

**Supplementary Table 2** The association of ambient air pollutants and intracerebral hemorrhage risk in time-stratified case-crossover design and symmetric bidirectional case-crossover design (OR, 95% CI)

|  | Time-stratified case-crossover | Symmetric bidirectional  case-crossover |
| --- | --- | --- |
| PM_2.5_ |  |  |
| Year: 2014-2017 | | |
| Lag 0 | 0.989 (0.964, 1.015) | 0.985 (0.960, 1.011) |
| Lag 1 | 0.989 (0.963, 1.016) | 0.991 (0.964, 1.019) |
| Lag 2 | 1.000 (0.975, 1.026) | 1.001 (0.973, 1.029) |
| Lag 3 | 1.009 (0.984, 1.034) | 1.000 (0.973, 1.028) |
| Lag 4 | **1.026 (1.001, 1.051) *** | 1.025 (0.998, 1.054) |
| Lag 5 | 1.017 (0.991, 1.043) | 1.017 (0.989, 1.047) |
| Year: 2018-2021 | | |
| Lag 0 | 0.995 (0.962, 1.030) | 0.992 (0.955, 1.032) |
| Lag 1 | 0.972 (0.938, 1.007) | 0.971 (0.934, 1.010) |
| Lag 2 | 0.974 (0.941, 1.008) | 0.966 (0.931, 1.003) |
| Lag 3 | 0.985 (0.953, 1.018) | 0.980 (0.946, 1.017) |
| Lag 4 | 0.991 (0.958, 1.025) | 0.980 (0.944, 1.018) |
| Lag 5 | 1.008 (0.975, 1.042) | 0.991 (0.955, 1.028) |
| PM_10_ |  |  |
| Year: 2014-2017 |  |  |
| Lag 0 | 0.990 (0.973, 1.006) | 0.989 (0.972, 1.006) |
| Lag 1 | 0.990 (0.973, 1.007) | 0.994 (0.976, 1.012) |
| Lag 2 | 0.997 (0.980, 1.014) | 0.997 (0.979, 1.015) |
| Lag 3 | 1.002 (0.985, 1.018) | 0.995 (0.977, 1.013) |
| Lag 4 | 1.013 (0.997, 1.029) | 1.014 (0.996, 1.033) |
| Lag 5 | 1.009 (0.993, 1.025) | 1.012 (0.993, 1.030) |
| Year: 2018-2021 |  |  |
| Lag 0 | 0.998 (0.974, 1.022) | 0.993 (0.967, 1.019) |
| Lag 1 | 0.983 (0.960, 1.007) | 0.981 (0.956, 1.007) |
| Lag 2 | 0.983 (0.961, 1.006) | 0.977 (0.952, 1.002) |
| Lag 3 | 0.997 (0.975, 1.020) | 0.993 (0.969, 1.018) |
| Lag 4 | 1.003 (0.981, 1.026) | 0.998 (0.973, 1.023) |
| Lag 5 | 1.009 (0.987, 1.032) | 1.000 (0.972, 1.025) |

***** *P*<0.05

**Abbreviations:** OR, odds ratio; CI, confidence interval

***Continued***

|  | Time-stratified case-crossover | Symmetric bidirectional  case-crossover |
| --- | --- | --- |
| SO_2_ |  |  |
| Year: 2014-2017 | | |
| Lag 0 | 1.005 (0.990, 1.020) | 1.004 (0.987, 1.021) |
| Lag 1 | 1.001 (0.985, 1.016) | 1.002 (0.985, 1.018) |
| Lag 2 | 1.004 (0.989, 1.020) | 1.003 (0.987, 1.019) |
| Lag 3 | 1.006 (0.990, 1.022) | 1.002 (0.985, 1.018) |
| Lag 4 | **1.020 (1.005, 1.035) *** | **1.020 (1.004, 1.036) *** |
| Lag 5 | 1.013 (0.997, 1.029) | **1.017 (1.000, 1.035) *** |
| Year: 2018-2021 | | |
| Lag 0 | 0.988 (0.943, 1.036) | 0.982 (0.934, 1.033) |
| Lag 1 | **0.947 (0.902, 0.994) *** | 0.957 (0.907, 1.008) |
| Lag 2 | 0.981 (0.936, 1.028) | 0.990 (0.941, 1.042) |
| Lag 3 | 1.010 (0.966, 1.056) | 1.003 (0.955, 1.053) |
| Lag 4 | 1.009 (0.965, 1.055) | 1.017 (0.967, 1.070) |
| Lag 5 | 1.017 (0.973, 1.063) | 1.023 (0.973, 1.075) |
| NO_2_ |  |  |
| Year: 2014-2017 |  |  |
| Lag 0 | 0.942 (0.886, 1.001) | **0.928 (0.869, 0.991) *** |
| Lag 1 | 0.956 (0.899, 1.016) | 0.952 (0.892, 1.017) |
| Lag 2 | 0.989 (0.931, 1.051) | 0.984 (0.921, 1.051) |
| Lag 3 | 1.020 (0.961, 1.082) | 1.028 (0.962, 1.099) |
| Lag 4 | 1.055 (0.997, 1.117) | **1.071 (1.005, 1.141) *** |
| Lag 5 | 1.048 (0.990, 1.110) | 1.061 (0.995, 1.131) |
| Year: 2018-2021 |  |  |
| Lag 0 | 0.990 (0.932, 1.052) | 0.984 (0.920, 1.052) |
| Lag 1 | 0.963 (0.905, 1.024) | 0.968 (0.906, 1.035) |
| Lag 2 | **0.918 (0.864, 0.976) *** | **0.921 (0.860, 0.986) *** |
| Lag 3 | 0.995 (0.937, 1.056) | 0.979 (0.916, 1.047) |
| Lag 4 | 0.989 (0.932, 1.049) | 0.966 (0.903, 1.033) |
| Lag 5 | 1.013 (0.955, 1.075) | 0.997 (0.932, 1.067) |

***** *P*<0.05

**Abbreviations:** OR, odds ratio; CI, confidence interval

***Continued***

|  | Time-stratified case-crossover | Symmetric bidirectional  case-crossover |
| --- | --- | --- |
| CO |  |  |
| Year: 2014-2017 | | |
| Lag 0 | 0.985 (0.955, 1.016) | 0.972 (0.939, 1.006) |
| Lag 1 | 0.998 (0.967, 1.029) | 0.991 (0.957, 1.026) |
| Lag 2 | 1.002 (0.971, 1.033) | 0.998 (0.964, 1.032) |
| Lag 3 | 1.020 (0.990, 1.051) | 1.016 (0.981, 1.053) |
| Lag 4 | **1.041 (1.012, 1.071) *** | **1.051 (1.017, 1.087) *** |
| Lag 5 | **1.036 (1.006, 1.066) *** | 1.028 (0.995, 1.063) |
| Year: 2018-2021 | | |
| Lag 0 | 0.999 (0.952, 1.048) | 0.993 (0.943, 1.045) |
| Lag 1 | 0.972 (0.925, 1.021) | 0.976 (0.926, 1.029) |
| Lag 2 | 0.962 (0.917, 1.009) | 0.959 (0.911, 1.009) |
| Lag 3 | 1.010 (0.965, 1.058) | 0.996 (0.948, 1.046) |
| Lag 4 | 0.990 (0.944, 1.037) | 0.974 (0.925, 1.026) |
| Lag 5 | 1.014 (0.968, 1.062) | 1.005 (0.955, 1.059) |
| O_3_ |  |  |
| Year: 2014-2017 |  |  |
| Lag 0 | 0.983 (0.954, 1.014) | 0.988 (0.956, 1.021) |
| Lag 1 | 0.980 (0.950, 1.010) | 0.986 (0.953, 1.019) |
| Lag 2 | 1.008 (0.979, 1.038) | 0.997 (0.966, 1.029) |
| Lag 3 | 1.001 (0.971, 1.031) | 1.013 (0.980, 1.047) |
| Lag 4 | 1.010 (0.980, 1.042) | 1.017 (0.983, 1.052) |
| Lag 5 | 1.017 (0.987, 1.048) | 1.005 (0.972, 1.040) |
| Year: 2018-2021 |  |  |
| Lag 0 | 1.028 (0.999, 1.058) | 1.019 (0.988, 1.051) |
| Lag 1 | 1.004 (0.976, 1.033) | 1.012 (0.981, 1.043) |
| Lag 2 | 1.001 (0.972, 1.030) | 1.002 (0.971, 1.033) |
| Lag 3 | 1.000 (0.972, 1.029) | 0.985 (0.955, 1.017) |
| Lag 4 | 0.990 (0.962, 1.019) | 0.990 (0.959, 1.022) |
| Lag 5 | 1.002 (0.974, 1.032) | 0.992 (0.962, 1.023) |

***** *P*<0.05

**Abbreviations:** OR, odds ratio; CI, confidence interval
